# An Experimental Exploration of Cognitive Workload and Situational Awareness in Virtual Reality: Implications for Non‐Clinical Emotional Support

**DOI:** 10.1002/mpr.70061

**Published:** 2026-01-28

**Authors:** Fabiha Islam, Zipporah Bright, Liang Zhan, Chao Shi

**Affiliations:** ^1^ School of Systems Science and Industrial Engineering Binghamton University Binghamton New York USA; ^2^ Electrical and Computer Engineering University of Pittsburgh Pittsburgh Pennsylvania USA

**Keywords:** cognitive workload, mental health, physiological measure, virtual reality

## Abstract

**Objectives:**

Many people face emotional challenges without meeting the criteria for clinical mental disorders. Virtual Reality (VR) has become popular in psychotherapy due to accessibility, but it can sometimes increase cognitive workload (CWL) and decrease situational awareness (SA). This study addresses the need for continuous user monitoring by assessing CWL and SA in real‐time using eye‐tracking.

**Methods:**

Twenty‐one participants performed cognitive tasks of varying difficulty in both virtual and real‐world environments, allowing direct comparison of the same task difficulty across the two environments. Pupil diameter, fixation, and saccade duration data were collected.

**Results:**

Pupil diameter results showed that VR environment may be associated with a reduced effect of high task difficulty on participants' CWL. Fixation and saccade durations results indicated that participants could maintain the same level of SA and engagement in the VR environment regardless of the task difficulty.

**Conclusion:**

These findings provide insights into the effects of VR on CWL and SA, which may inform future research exploring VR's potential in clinical populations.

## 1 Introduction

Untreated emotional challenges and mild depression can escalate into more severe forms of mental disorders, increasing healthcare costs and diminishing quality of life. Mental disorders, also referred to as mental health conditions, involve significant disruptions in an individual's cognitive processes, emotional regulation, or behavior, often leading to distress or impairments in essential areas of daily functioning (World Health Organization [WHO] 2022). Despite growing knowledge about the causes of mental disorders and increasing access to specialized treatment, the prevalence of these conditions remains alarmingly high (Cieślik et al. 2020). For example, in 2019, approximately 1 in 8 individuals globally, or about 970 million people, were living with a mental disorder, with anxiety and depression being the most prevalent. The onset of the COVID‐19 pandemic in 2020 further exacerbated these numbers, leading to a significant increase in cases of anxiety and depressive disorders (WHO 2022). The severity of mental disorders can vary widely, with some individuals able to manage daily life while others may be disabled by their illness (Mozafaripour 2024). Common examples of such disorders include anxiety, depression, schizophrenia, bipolar disorder, eating disorders, alcohol use disorders, autism spectrum disorder, conduct disorder, and dysthymia (Mozafaripour 2024; Wu et al. 2023). Numerous barriers to effective treatment exist, including stigmatization, financial constraints, geographic inaccessibility, difficulty securing appointments, dissatisfaction with services, a lack of culturally competent care, and reluctance to report symptoms or seek help (Andrade et al. 2014). The societal burden of mental disorders is significant, including not only the direct costs associated with diagnosis and treatment but also indirect costs such as productivity losses, early retirement, disability, and reduced quality of life (Wiebe et al. 2022; Wu et al. 2023). Furthermore, individuals with mental disorders are often subjected to human rights violations, discrimination, and stigma, which can worsen their condition (Wu et al. 2023). In response to these challenges, efforts are increasingly directed toward the development of digital therapeutics (DTx) and other healthcare technologies, such as telemedicine, healthcare apps, and virtual reality (VR), aiming to expand access to evidence‐based treatments and reduce healthcare costs (Patel and Butte 2020; Wiebe et al. 2022). VR, in particular, is emerging as a promising tool to enhance psychological well‐being by facilitating new learning and therapeutic intervention (Cieślik et al. 2020).

Mental support in technology has focused on patients with mental disorders, while ignored the requirements of general population with non‐clinical emotional challenges. Over the years, technologies have played an essential role in supporting mental healthcare by facilitating the screening, diagnosis, and treatment of mental health conditions while promoting overall well‐being. Among these technologies Virtual Reality (VR) has gained massive popularity in recent ages due to its unique ability to overcome the barriers of conventional mental health support systems. VR serves as a technological interface which is capable of immersing users in a computer‐generated three‐dimensional environment that mimics the real‐world scenarios. By utilizing wearable head mounted displays (HMDs), noise cancellation headsets, gesture‐sensing gloves, synthesized sounds, and haptic platforms, users can engage in an interacting experience with an enhanced sense of presence (Colombo et al. 2022; Dilgul et al. 2021; Freeman et al. 2017; Maples‐Keller et al. 2017; Pons et al. 2022; Valmaggia et al. 2016; Zhai et al. 2021). VR's capability to regulate and manipulate exposure doses and stimuli offers precise control of clinical and therapeutics settings, helping individuals to manage mental challenges by eliciting targeted emotional responses. The spatial representation of VR facilitates visualizing and enhancing the vividness of fictional activities, which would be challenging to achieve through imagination alone (Colombo et al. 2022). The experiences offered by VR are repeatable, testable, adaptable, and difficult to recreate in real life, thus making it a powerful tool for the psychiatric community while enhancing treatment consistency (Freeman et al. 2017; Maples‐Keller et al. 2017; Valmaggia et al. 2016). Although VR demonstrates vast potential to enhance psychotherapeutic interventions for mental disordered populations, a thorough investigation of its efficacy and impact on non‐clinical people's internal states such as perceived workload and attentional requirements has not yet been conducted. There is a need to assess the effect of VR on non‐clinical people who need emotional support.

VR has shown to be a promising but challenging tool in healthcare treatments. Recent advancements of software and hardware devices, along with the availability of affordable consumer HMDs, have facilitated the widespread application of VR in mental healthcare (Baghaei et al. 2021; Colombo et al. 2022). It has demonstrated effectiveness in treating a broad spectrum of psychological conditions, including anxiety disorders, stress disorders, panic disorder, obesity, binge eating disorders, pain management, and schizophrenia (Baghaei et al. 2021; Colombo et al. 2022; Dilgul et al. 2021; Srivastava et al. 2014; Zhai et al. 2021). Additionally, VR has been applied in counseling and cognitive‐behavioral therapy to target addiction and specific phobias, including acrophobia, spider phobia, agoraphobia, social phobia, claustrophobia, trypanophobia, astraphobia, fear of flying, and fear of driving. It has also been used in cognitive rehabilitation programs to help autistic individuals acquire essential skills (Srivastava et al. 2014). Despite the extensive application and advantages of VR, people have reported limitations and challenges that include hardware and software malfunction, setup difficulties, as well as cyber sickness symptoms such as dizziness, headache, eye fatigue, reduced limb control and disorientation, nausea, and inappropriate real‐world responses. These factors can affect the users with a potential increase or decrease of their cognitive workload (CWL) and impact their efficacy within virtual world (Chang et al. 2020; DAŞDEMİR 2022; Dong et al. 2021; Drouot et al. 2022; Islam et al. 2024; Maples‐Keller et al. 2017; Srivastava et al. 2014; Xi et al. 2023). Assessment of the CWL within a VR environment can assist in avoiding the risk of overwhelming the users and adversely affecting their task performance. Additionally, limited evidence exists regarding the application of VR in treating people who experience emotional challenges while minimizing patients' CWL. Therefore, in this study we focus on evaluating individuals' CWL while they perform mentally challenging tasks in a VR environment and establishing a baseline to aid future researchers in personalizing VR environments based on patients' preference, interest, and mental condition. Subsequent research will consider inclusion of individuals with mental disorder within a virtual environment to measure its efficacy and impact on their mental condition.

CWL can be defined as a physiological change in the visceral motor system that requires the contribution of mental effort to cope with the task (Islam et al. 2023; Reiner and Gelfeld 2014). The use of physiological indices such as eye‐tracking within virtual environment offers several advantages that include continuous and real‐time mental states monitoring, avoidance of interference with the primary task during data collection, non‐intrusiveness, and the provision of objective evaluations (Das et al. 2020; Drouot et al. 2022; Markopoulos et al. 2021; Reiner and Gelfeld 2014; Tao et al. 2019). Among several eye‐tracking metrics, pupil diameter and fixation duration are the most widely used parameters to assess individuals' attentional states. Several studies have shown that pupil diameter demonstrates strong correlation with CWL of an ongoing processing while fixation duration serves as a sensitive indicator of SA during task performance (Argyle et al. 2020; Chien et al. 2015; Kim and Kim 2020; Pfleging et al. 2016; Salehi et al. 2018). It has been reported that a higher level of cognitive demanding tasks creates a higher mental workload which generates larger pupil diameter (Di Stasi et al. 2011; Dong et al. 2021; Gao et al. 2021; Medathati et al. 2020; Menekse Dalveren et al. 2018; Reiner and Gelfeld 2014; Tao et al. 2019). Salehi et al. (2018) found that experts had longer fixation durations and higher fixation numbers in situations of significant drill pipe pressure variation than non‐experts, indicating better SA. In the area of healthcare, Desvergez et al. (2019) found that experienced anesthetists on average had more fixation numbers and longer total fixation durations than novices, again indicating better SA. Another widely used eye‐tracking index is saccade, a rapid eye movement between two consecutive fixations (Lai et al. 2013). The duration of saccades has been used to measure attentiveness and risk perception (Shadiev and Li 2023; Yang et al. 2021). Though visual behaviors are valuable indicators of mental states, the relationship between these measures and the mental state depends on several factors such as task type, environment, and biological factors. In this study, pupil diameter, fixation duration, and saccade duration were used to measure CWL while performing mentally demanding tasks at different difficulty levels within a real as well as virtual environment.

The objective of this study is to measure people's CWL and SA using eye‐tracking measures in a VR environment and compare that with real‐world settings. This study is innovative in that it will track human internal states in real‐time in a VR environment for mental healthcare purposes, which is very rare. We hypothesize that there will be significant differences between the eye‐tracking metrics measured within the real‐world and VR environments. Accordingly, our current study takes the form of two (VR vs. real‐world) times two (easy vs. difficult) trials within subject experiments where the subjects perform a mental load demanding task. During the task, participants' eye‐tracking data were collected using HMD and eye‐tracking goggles while the subject measure of workload was collected using NASA Task Load Index (NASA‐TLX) (Hart and Staveland 1988) after each session.

## 2 Methodology

### 2.1 Sample Size Calculation

To calculate an appropriate sample size, an a priori power analysis was applied using G*Power software (version 3.1.9). For a repeated measures within factors ANOVA, the required sample size is determined based on the partial eta squared (*η* ^2^) value for the interaction between the factors, such as environments and task difficulty levels. In this study, data from a pilot study with six participants were used to estimate the effect size (based on *η* ^2^ = 0.332), the correlation among repeated measures, and the Nonsphericity correction factor (*ε*). Power analysis with an alpha (*α*) level of 0.05 and a power (1‐β) level of 0.8 indicated that a minimum sample size of five participants would be sufficient to detect within‐subjects effects. Nevertheless, twenty‐one participants were recruited to enhance the reliability and robustness of the results.

### 2.2 Participants

We recruited 21 right‐handed college students without any clinical mental condition (18 male, 3 female, aged 18–35) to participate in this study, regardless of their prior VR experience. None of the participants had any current mental health conditions or prior psychotherapy experience. All the participants had normal or corrected‐to‐normal eyesight. Individuals with current musculoskeletal disorders and visual impairment that was not correctable with lenses were excluded from the experiment. Table 1 shows the mean age of the male and female participants along with the standard deviation (Std).

**TABLE 1:** Mean age (std) of the participants.

Gender	No. of participant	Mean age (std)	Race/ethnicity
Male	18	22.56 (2.26)	White (*n* = 17), asian (*n* = 3), black (*n* = 1)
Female	3	22.33 (5.13)	
Total	21	22.57 (2.56)	

Abbreviations: *n* = number of participants; Std = standard deviation.

### 2.3 Apparatus

The study utilized several software and hardware devices to perform the experimental procedure.

#### 2.3.1 Cognitive Load Demanding Task

In the experiment, we used Fruit Ninja 2 as the cognitive‐load‐demanding task because it is engaging, easy to play, and can be readily adapted to both VR and physical real‐world settings. Participants played the game by slicing the fruits that appeared while avoiding the bombs presented during gameplay. Fruit Ninja 2 was administered at two difficulty levels in both environments: Arcade and Classic. The Arcade mode served as the easy condition, requiring players to slice as many fruits as possible within 1 minute while avoiding bombs. In contrast, the Classic mode represented the more challenging condition, as players were required to slice every fruit that appeared while still avoiding bombs. This game shares conceptual similarities with psychotherapy, as both involve cognitive processing, active engagement with one's thoughts and actions, distraction from negative thinking, and the opportunity to experience a sense of accomplishment.

#### 2.3.2 Real‐World Setting

**FIGURE 1:**
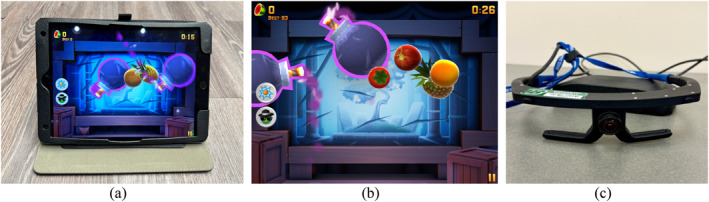
The equipment used in the real‐world setting: (a) Apple iPad (9th generation) used for the gameplay, (b) Fruit Ninja 2 interface presented to participants, (c) Argus Science glasses (ETVision) to record eye tracking data.

In the real‐world setting, the task was presented on an Apple iPad (9th generation) (Figure 1a). Fruit Ninja 2 was downloaded from the Apple App Store and installed on the device before the experiment. During the task, participants viewed the game directly on the iPad screen (Figure 1b) and interacted with it using touch gestures, slicing the on‐screen fruits with their fingers as they appeared. In the real‐world condition, participants' eye tracking data were recorded using the Argus Science glasses from ETVision (Figure 1c) while they played the game on an iPad.

#### 2.3.3 VR Settings

**FIGURE 2:**
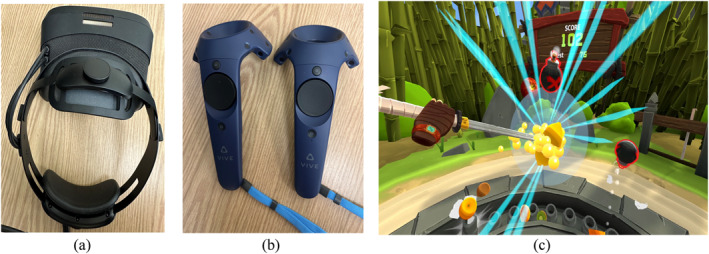
The equipment used in the VR setting: (a) Varjo VR‐3 HMD used to project the task and collect eye tracking data, (b) HTC Vive controllers used to interact with the VR environment, (c) projection of fruit ninja 2 in the VR setting.

In the VR setting, the task was projected using a Varjo VR‐3 HMD (Figure 2a). The Steam VR application was used to display the game within the VR environment. During the task, participants viewed the game in an immersive VR environment and interacted with it using two HTC Vive controllers (Figure 2b). In the VR condition, participants' eye tracking data were recorded using the Varjo VR‐3 HMD while they played the game in the immersive VR environment (Figure 2c).

#### 2.3.4 Self‐Reported Survey

We used NASA‐TLX (Hart and Staveland 1988) to collect participants' subjective CWL after performing the task. NASA‐TLX is a multidimensional survey‐based measure that collects subjective feedback on mental, physical, and temporal demand, frustration, effort, and performance. Participants rated each of the six subscales of NASA‐TLX on a bipolar scale and completed fifteen paired comparisons to determine the relative weight of each subscale. The overall CWL score was calculated as the weighted mean of the six dimensions, ranging from 0 to 100, with lower scores indicating lower CWL and higher scores indicating higher CWL.

### 2.4 Experimental Procedure

**FIGURE 3:**
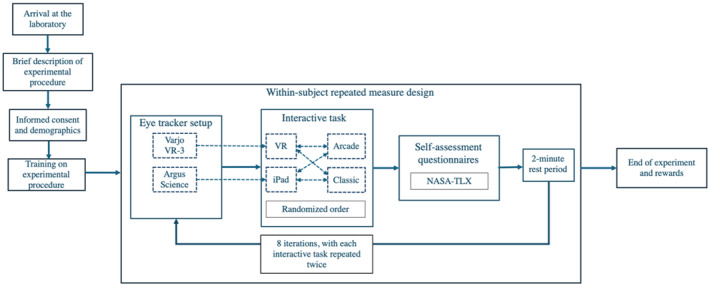
Schematic presentation of the experimental procedure.

The study was approved by the Institutional Review Board of Binghamton University, and recruitment materials were distributed both physically and virtually across the university. The inclusion criteria required participants to be adults aged 18–35, with or without prior virtual environment experience, and to have normal vision or be able to achieve it by wearing contact lenses during the experiment. Exclusion criteria included individuals with visual impairments or myopia requiring only glasses, who could not wear contact lenses, and those with current musculoskeletal injuries or severe motion sickness. Interested individuals who contacted the researcher were invited to the laboratory after an initial eligibility screening. Upon arrival, the experimental procedure was briefly described to the participants, and signatures were obtained on the informed consent form. Before the study, demographic data, including age, gender, race/ethnicity, and hand dominance, were collected from the participants. Participants were also asked about any prior psychotherapy experience and whether they had any current mental health conditions, including both clinically diagnosed and self‐identified conditions. Next, they were provided with a training on the experimental process for approximately 10 min. During the training session, participants received step‐by‐step guidance on the setup, activation, and proper placement of the Argus Science glasses and the Varjo VR‐3 headset. Detailed instructions were provided for the correct use of the glasses and headset, including adjusting the headband for a secure, comfortable fit and optimizing visual clarity. Participants were also trained in the operation of the VR controllers, including the functions and layouts of each button, to ensure confident interaction with the virtual environment. Finally, they were briefed on the rules and specific task requirements for the Fruit Ninja 2 game scenarios, allowing them to familiarize themselves with the gameplay procedures and interactions before the experimental tasks. Once the participants were comfortable operating the games individually, they were asked to play the Fruit Ninja 2 game in two environments (real‐world and VR) at two difficulty levels (easy and hard) with repeated measures of the factors. The order of real‐world and VR trials was randomized for each participant to avoid the order effect. Following the completion of each trial, participants were asked to complete a self‐assessment questionnaire, using the electronic version of the NASA‐TLX, to report their perceived CWL during the task. To reduce mental fatigue and ensure consistent performance, a two‐minute rest period was provided after each gameplay and questionnaire session. Upon completion of the entire experimental procedure, which lasted approximately 90 min, participants were compensated with a $15 gift card. Figure 3 illustrates the step‐by‐step experimental procedure employed in this study.

### 2.5 Data Analysis

In this study, each participant experienced eight trials; as a result, 21 participants yielded 168 (21 ×8) trials. Eye‐tracking measurements such as left‐pupil diameter, fixation duration, and saccade duration were collected and analyzed. ETAnalysis software and similar algorithms were used to extract pupil diameter (between 2 and 8 mm), fixation duration (larger than 100 ms), and saccade duration (between 20 and 60 ms) from the eye‐tracking dataset measured in the real and VR environment, respectively. To assess participants perceived CWL, the overall mental workload collected by NASA‐TLX was considered. To prevent participants from feeling pressured to achieve higher scores, which could elevate their workload, no performance requirements were imposed as long as no obvious maladaptive behaviors were observed. Consequently, game performance and scores were not recorded. A list of independent and dependent variables is shown in Table 2.

**TABLE 2:** Independent and dependent variables.

Independent variable	Dependent variable
Environment • VR • Real‐world	Eye‐tracking data • Left‐pupil diameter (mm) • Fixation duration (ms) • Saccade duration (ms)
Difficulty level • Easy • Hard	NASA‐TLX

Abbreviations: mm = millimeters; ms = milliseconds; NASA‐TLX = nasa task load index; VR = virtual reality.

Subsequent data analyzed was carried out using IBM Corporation's Statistical Package for Social Science (SPSS) version 28. The Anderson‐Darling test was used to assess data normality. As the data remained non‐normal (*p* < 0.005) even after the Johnson transformation, the Wilcoxon Signed‐Rank nonparametric test was applied to examine differences in pupil diameter, fixation duration, and saccade duration across environments and task difficulty levels. Four groups of independent variables were formed: real‐world easy; real‐world hard; VR easy; and VR hard. Results were considered statistically significant at *p* < 0.05. The test was conducted with six combinations of the groups: real‐world easy to real‐world hard; real‐world easy to VR easy; real‐world easy to VR hard; real‐world hard to VR easy; real‐world hard to VR hard; VR hard to VR easy. The analysis with a Bonferroni correction yielded a significance level of 0.008 (=0.05/6). Finally, a Pearson Correlation analysis was conducted between NASA‐TLX and pupil diameter to test the relationship between the subjective and objective measurements.

## 3 Results

### 3.1 Pupil Diameter

**FIGURE 4:**
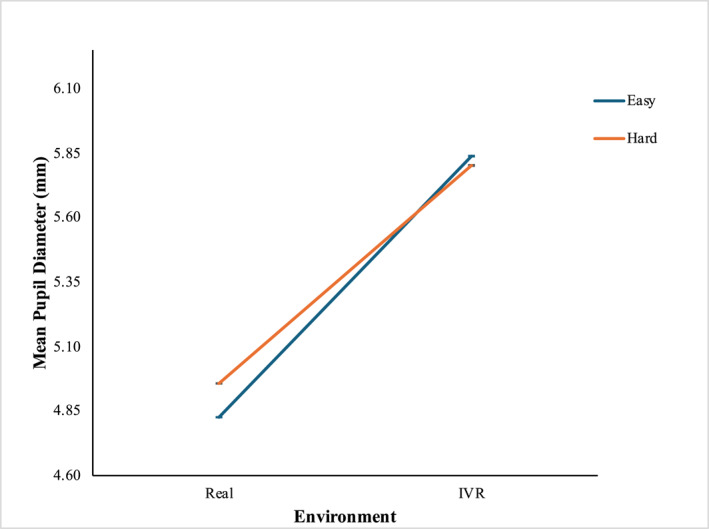
Mean pupil diameter (mm) at four groups of independent variables.

The Wilcoxon Signed‐Rank test result demonstrated statistically significant differences in the pupil diameter measurements obtained from the four groups of independent variables. The analysis with Bonferroni correction showed that all four groups were significantly different from each other in terms of pupil diameter (Figure 4). Mean pupil diameter in VR was significantly higher than in the real‐world environment at the easy level ($M_{VR}$ = 5.84 mm, ${SD}_{VR}$ = 0.96, and $M_{Real}$ = 4.83 mm, ${SD}_{Real}$ = 0.64; *Z* = −491.525, *p* < 0.001) and the hard level ($M_{VR}$ = 5.80 mm, ${SD}_{VR}$ = 0.75, and $M_{Real}$ = 4.96 mm, ${SD}_{Real}$ = 0.75; *Z* = −286.471, *p* < 0.001). In the real‐world environment, mean pupil diameter was significantly higher at the hard level than at the easy level ($M_{Hard}$ = 4.96 mm, ${SD}_{Hard}$ = 0.75, and $M_{Easy}$ = 4.83 mm, ${SD}_{Easy}$ = 0.64; *Z* = −4.975, *p <* 0.001) while the result is the opposite in the VR environment ($M_{Hard}$ = 5.80 mm, ${SD}_{Hard}$ = 0.75, and $M_{Easy}$ = 5.84 mm, ${SD}_{Easy}$ = 0.96; *Z* = −16.892, *p <* 0.001). The mean left pupil diameter with standard deviation (std) and 95% confidence interval (CI) are shown in Table 3.

**TABLE 3:** Mean (Std) and 95% CI of left pupil diameter, fixation duration, and saccade duration.

Eye‐tracking measures	Groups of environment and difficulty level	Mean (std)	95% CI	
			Lower bound	Upper bound
Left pupil diameter	Real‐easy	4.83 (0.64)	4.82	4.83
	Real‐hard	4.96 (0.75)	4.96	4.96
	VR‐easy	5.84 (0.96)	5.84	5.84
	VR‐hard	5.80 (0.75)	5.80	5.81
Fixation duration	Real‐easy	176.86 (0.11)	175.85	177.88
	Real‐hard	175.65 (0.11)	174.73	176.56
	VR‐easy	169.11 (0.13)	167.02	171.2
	VR‐hard	172.17 (0.11)	169.24	175.11
Saccade duration	Real‐easy	33.94 (9.42)	33.65	34.23
	Real‐hard	34.59 (9.89)	34.32	34.85
	VR‐easy	37.46 (11.81)	34.01	40.9
	VR‐hard	39.78 (12.05)	36.18	43.39

Abbreviations: CI = confidence interval; Std = standard deviation; VR = virtual reality.

### 3.2 Fixation Duration

**FIGURE 5:**
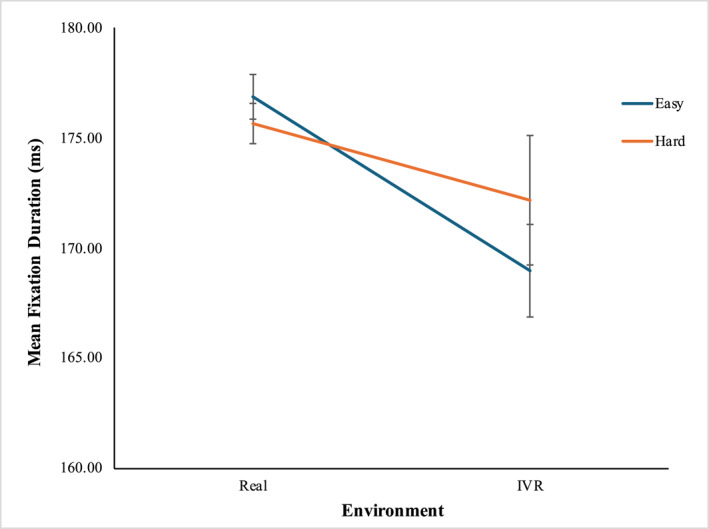
Mean fixation duration (ms) at four groups of independent variables.

Wilcoxon Signed‐Rank test result demonstrated statistically significant differences in the mean fixation durations measured within the four groups of independent variables (Figure 5). Mean fixation duration in VR was significantly lower than in the real‐world environment at the easy level ($M_{VR}$ = 169.11 ms, ${SD}_{VR}$ = 0.13, and $M_{Real}$ = 176.86 ms, ${SD}_{Real}$ = 0.11, *Z* = −3.877, *p* < 0.001), while no significant difference was observed at the hard level. In the real‐world environment, the mean fixation duration was significantly higher at the easy level than at the hard level ($M_{Easy}$ = 176.86 mm, ${SD}_{Easy}$ = 0.11, and $M_{Hard}$ = 175.65 mm, ${SD}_{Hard}$ = 0.11; *Z* = −2.719, *p* = 0.007) while no significant difference was observed in the VR environment. The mean fixation durations with std and 95% CI are shown in Table 3.

### 3.3 Saccade Duration

**FIGURE 6:**
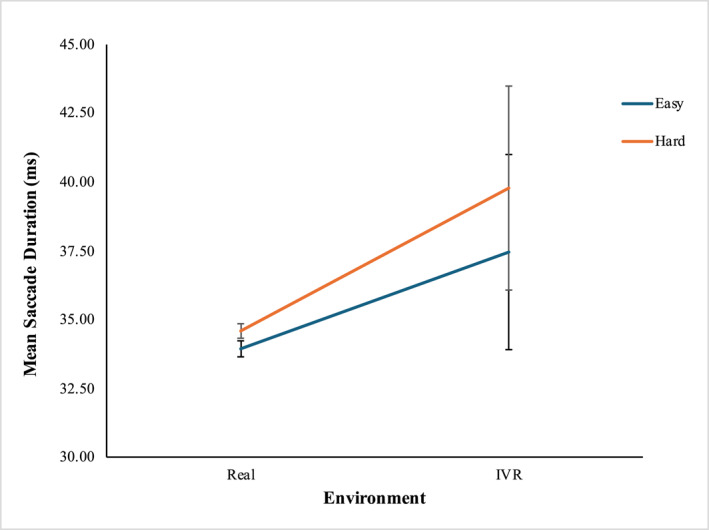
Mean saccade duration (ms) at four groups of independent variables.

From the Wilcoxon Signed‐Rank test result, it was found that statistically significant differences existed between the saccade durations observed in the four groups of independent variables (Figure 6). Mean saccade duration in VR was significantly higher than in the real‐world environment at the easy level ($M_{VR}$ = 37.46 ms, ${SD}_{VR}$ = 11.81, and $M_{Real}$ = 33.94 ms, ${SD}_{Real}$ *D* = 9.42; *Z* = −2.929, *p* = 0.003), while no significant difference was observed in hard level. In the real‐world environment, the mean saccade duration was significantly higher at the hard level than at the easy level ($M_{Hard}$ = 34.59 mm, ${SD}_{Hard}$ = 9.89, and $M_{Easy}$ = 33.94 mm, ${SD}_{Easy}$ = 9.42; *Z* = −4.015, *p* < 0.001), while no significant difference was observed in the VR environment. The mean saccade durations with std and 95% CI are shown in Table 3.

### 3.4 Correlation Analysis

**FIGURE 7:**
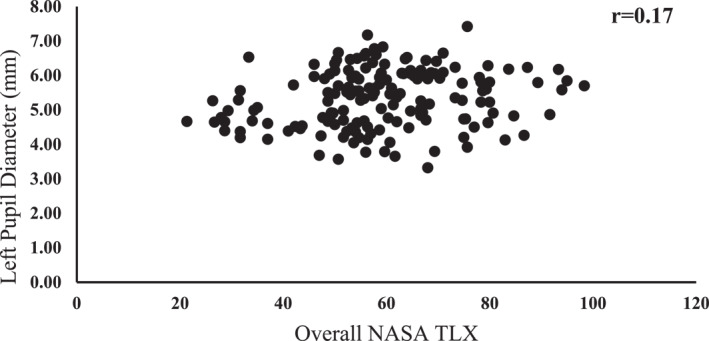
Correlation between left pupil diameter and overall NASA TLX.

The correlation analysis revealed a significant correlation between the pupil diameter and the overall CWL (*r* = 0.17, *p* = 0.029) (Figure 7). While the magnitude of the correlation coefficient suggests a weak relationship, the positive correlation implies that the pupil diameter increases as the overall CWL increases.

## 4 Discussion

In this study, individuals' mental workload while performing cognitive load demanding task in virtual and real‐word environment was explored. Physiological measure, that is, eye‐tracking data were collected from 21 participants in both environments to measure and compare their CWL and SA. Additionally, NASA‐TLX was used to collect their perceived mental workload and validate the findings of physiological measurements. Although the study did not include a clinical population, the results offer explanatory insights that could motivate future research on exploring the potential applications of VR in mental health–related interventions.

The results indicated that both environmental conditions and task difficulty levels had significant effects on participants' left pupil diameter during the Fruit Ninja 2 game. Across both difficulty levels, average pupil diameter was consistently larger in the virtual environment than in the real‐world condition. This dilation in pupil size may suggest that the concurrent CWL was significantly higher when participants played the game in the immersive virtual world compared to the real‐world environment. The observed variation in pupil diameter across environments could be influenced by the differences in illumination, as pupil size is susceptible to lighting conditions, leading to changes of several millimeters (Pfleging et al. 2016). These results indicate that participants were more engaged and focused on the game when presented in the virtual environment and at a higher difficulty level (Gao et al. 2021). In contrast, with its more naturalistic viewing conditions, the real‐world environment may have elicited a reduced cognitive workload (Medathati et al. 2020). Additionally, in the VR environment, pupil size was significantly larger during easy level compared to hard difficulty level, suggesting that while VR induces a higher cognitive workload overall, the increase in workload with rising task difficulty may be less pronounced under VR conditions. This characteristic of VR could be particularly advantageous for individuals experiencing emotional challenges, as it may reduce the mental strain associated with stressful situations.

Our correlation analysis further showed a positive relationship between the pupil size and perceived CWL, which supports the idea that physiological measures such as pupil diameter are sensitive to changes in mental workload and can serve as noninvasive, real‐time indicators of CWL. This relationship further indicates that individuals experience increased CWL in a VR environment, particularly at higher task difficulty levels.

After analyzing the fixation duration, it was found that while playing the game at the easy level, the mean fixation duration observed in the real‐world was significantly longer than the mean fixation duration in virtual settings. Fixation duration, which measures the time spent focusing on areas of interest, provides insight into individuals' tendencies to concentrate on critical points. Previous research indicates that longer fixation duration is directly proportional to CWL and SA (Salehi et al. 2018; Argyle et al. 2020). The mean fixation duration illustrates the extent of cognitive processing that includes interpreting and assessing visual information, as well as the level of attentional focus deployment (Chang et al. 2021; Chien et al. 2015; Dong et al. 2021; Kim and Kim 2020; Marquart et al. 2015). Based on these, our findings suggest that participants required extended attention and found it more difficult to decode visual information when performing the task in the real‐world environment compared to the VR environment (Salehi et al. 2018). One plausible explanation for this circumstance could be the VR's ability to project instantaneous panoramic streaming, which assisted the participants to easily recognize and process the visual information. The shorter duration of fixation observed in the virtual environment could be the result of cybersickness associated with the immersive virtual world (Chang et al. 2021). Additionally, longer fixation durations in the real‐world environment suggest that participants used the real‐world projection to establish a more comprehensive situational understanding and had a better SA compared to VR (Argyle et al. 2020). We also found that when the game was played in a real‐world environment, the mean fixation durations at the easy level were significantly higher than the hard level, while no difference was observed between the two difficulty levels in virtual world. This finding suggests that the level of easiness in real world scenario resulted in mitigated cognitive processing, which encouraged the participants to be more relaxed, extend their focus period, and develop a more comprehensive situational picture (Argyle et al. 2020). Additionally, the lack of significant differences between the fixation durations of two difficulty levels in the VR environment indicates that VR has the potential to maintain SA in various task difficulty levels, while in the real‐world environment, the SA decreases as the task difficulty increases.

We found that when the game was played in the real‐world environment, the mean saccade duration for hard level was significantly higher compared to the mean saccade duration of easy level. The higher difficulty level of the task requires greater attention and increased involvement of cognitive resources from the participants. The longer duration of saccade at higher difficulty level may arise from the elevated cognitive load as a way of allowing the participants to take more time to process information and make decisions. Besides, at the easy level of the game, the mean saccade duration noticed in the virtual world was higher compared to the real‐world environment. The longer saccade duration of VR environment can be contributed to individuals' engagement with complicated and dynamic visual presentation, immersiveness, and cognitive challenges associated with the virtual world. Contrary to our findings, Das et al. (2020) reported a significant negative correlation between mental demand and saccade duration. Their study found that shorter saccade durations were linked to heightened mental workload, and this increased mental workload was associated with the experience of hazards among operators in a virtual environment simulating electric overhead traveling crane operations (Das et al. 2020). The discrepancy in findings may be attributed to the fact that physiological data can vary significantly based on individual participants' physiology, differing responses to stimuli, and task types. An interesting finding from our study is that in the VR environment, no significant difference was observed in saccade durations between tasks of easy and hard difficulty levels, while in the real‐world environment, significant difference was noticed between the mean saccade durations for these difficulty levels. This suggests that saccade duration my remain consistent regardless of task difficulty in VR, which may imply that VR may help maintain user engagement even though the task difficulty level increases. In contrast, in the real world, engagement appears to decrease as task difficulty increases.

In summary, the findings suggest that although the perceived CWL within the virtual environment surpasses that of the traditional realistic environment, VR has the potential to encourage its users to be more attentive and focused to the tasks. Additionally, the realistic projection of environmental components within the VR could assist in decoding and processing the visual information with optimal focus. VR may be able to alleviate the impact of increased task difficulty and be associated with more consistent user engagement as task difficulty increases. It is obvious that the availability of abundant, yet easily accessible resources, coupled with the anonymity of the virtual realm may encourage the individuals to comfortably share their stories with professionals and others with the same issues (Usmani et al. 2022). Therefore, when designing VR tasks, careful consideration is needed to tailor devices required to enter the VR, as well as the virtual environment design. It is essential to consider the level of engagement and visual complexity in VR environments, as overly demanding or complicated presentations may increase cognitive load or discomfort. Additionally, VR exposure could also contribute to cybersickness, particularly for users with mental health conditions. Using virtual avatars or simplified representations, rather than fully immersive 3D environments, may help reduce these challenges. These findings of this study encourage us to explore the inclusion of individuals with mental health conditions in subsequent research to evaluate the potential of VR in mental health treatment.

Though the results of this study provide valuable information, several limitations have to be acknowledged. First, the findings from this study can be generalized only for the tasks that exhibit similar organizational structure, mental workload requirements, and consistency in visual search behavior. Secondly, the participants of this study were exposed to the virtual environment for a brief period. Extended exposure may affect the CWL and attentional requirements of the participants and potentially provide more insights. A third limitation of the study is the potential impact of varying light conditions, which was not treated as an independent variable in this study. The experiment employed different devices, such as an iPad for real‐world situations and an HMD for virtual conditions. These lighting or luminance disparities may have influenced the observed differences in pupil diameter. Future studies could consider the impact of different lighting conditions on eye tracking data of the users. Fourth, we used only eye‐tracking measures to understand the CWL, while there are several other physiological measures that can be used in the future to increase the validity and robustness of the assessment. Next, only young adults (aged 18–35) were recruited in this experiment. As the application of VR expands across various domains, it is anticipated that older adults will increasingly engage with virtual environments. Moreover, the gender imbalance among participants (18 males and 3 females) may limit the generalizability of the findings. Therefore, future research should aim to include a more diverse age range and achieve gender balance to better understand the cognitive workload and performance of a broader range of individuals within VR environments. Finally, this study included only individuals who reported having no current clinical or self‐reported mental health conditions. However, no structured clinical screening was conducted to verify participants' mental health status, including the absence of emotional symptoms or psychiatric disorders. Therefore, the findings from this sample should be interpreted with caution. Future research incorporating comprehensive assessments of participants' clinical status is needed to more accurately evaluate the effects of VR in individuals with and without mental health conditions.

## 5 Conclusion

In conclusion, this experiment provided insights into non‐clinical peoples' CWL within a real‐world and a VR environment at low and high difficulty level of a cognitively demanding task. Participants exhibited higher CWL and lower SA within VR compared to real‐world settings. However, VR's real‐time immersive projection facilitated efficient recognition and processing of visual information, may be associated with a reduced impact of increased task difficulty. Additionally, VR has the potential to maintain user engagement even as task difficulty increases. The study indicates that eye‐tracking measures could be used as a good indicator of CWL and attentional requirements, especially within a virtual environment while performing mentally challenging tasks. The findings of this study can inform future research on the design of VR environments aimed at optimizing CWL, SA, and user engagement. Additionally, these results may motivate subsequent studies to include clinical populations to explore the potential applications of VR in therapeutic contexts.

## Author Contributions

**Fabiha Islam:** methodology, software, data curation, investigation, validation, formal analysis, visualization, writing – original draft. **Zipporah Bright:** methodology, data curation. **Liang Zhan:** writing – review and editing. **Chao Shi:** conceptualization, investigation, validation, supervision, funding acquisition, project administration, resources, writing – review and editing.

## Funding

The authors have nothing to report.

## Ethics Statement

Ethical approval was obtained from the Institutional Review Board of Binghamton University prior to data collection. All participants provided informed consent before participating in the study and were free to withdraw at any time without penalty.

## Conflicts of Interest

The authors declare no conflicts of interest.

## Data Availability

The data that support the findings of this study are available from the corresponding author upon reasonable request.
